# Linking Intra-Articular Inflammatory Biomarkers with Peripheral and Central Sensitization in Late-Stage Knee Osteoarthritis Pain: A Pilot Study

**DOI:** 10.3390/jcm13175212

**Published:** 2024-09-02

**Authors:** Sofie Puts, Rose Njemini, Thomas Bilterys, Nina Lefeber, Thierry Scheerlinck, Jo Nijs, David Beckwée, Ivan Bautmans

**Affiliations:** 1 Gerontology Department, Vrije Universiteit Brussel, Laarbeeklaan 103, 1090 Brussels, Belgium; sofie.puts@vub.be (S.P.);; 2 Frailty & Resilience in Ageing (FRIA) Research Unit, Vitality Research Group, Vrije Universiteit Brussel, Laarbeeklaan 103, 1090 Brussels, Belgium; david.beckwee@vub.be; 3 Pain in Motion Research Group (PAIN), Vrije Universiteit Brussel, Laarbeeklaan 103, 1090 Brussels, Belgium; thomas.bilterys@vub.be (T.B.); jo.nijs@vub.be (J.N.); 4 Rehabilitation Research Group (RERE), Vrije Universiteit Brussel, Laarbeeklaan 103, 1090 Brussels, Belgium; 5 Department of Orthopedic Surgery and Traumatology, Universitair Ziekenhuis Brussel, Laarbeeklaan 101, 1090 Brussels, Belgium; thierry.scheerlinck@uzbrussel.be; 6 Department of Physiotherapy, Human Physiology and Anatomy, Faculty of Physical Education and Physiotherapy, Vrije Universiteit Brussel, Laarbeeklaan 103, 1090 Brussels, Belgium; 7 Department of Physical Medicine and Physiotherapy, Universitair Ziekenhuis Brussel, Laarbeeklaan 101, 1090 Brussels, Belgium; 8 Unit of Physiotherapy, Department of Health and Rehabilitation, Institute of Neuroscience and Physiology, Sahlgrenska Academy, University of Gothenburg, 40530 Gothenburg, Sweden; 9 Research Group MOVANT, Department of Rehabilitation Sciences and Physiotherapy (REVAKI), University of Antwerp, Universiteitsplein 1, 2610 Wilrijk, Belgium; 10 Geriatrics Department, Universitair Ziekenhuis Brussel, Laarbeeklaan 101, 1090 Brussels, Belgium; 11 Department of Geriatric Physiotherapy, SOMT University of Physiotherapy, Softwareweg 5, 3821 BN Amersfoort, The Netherlands

**Keywords:** osteoarthritis, sensitization, knee arthroplasty, synovial fluid, pain, inflammation

## Abstract

**Background/Objectives**: To investigate if intra-articular biomarkers relate to peripheral and central sensitization in patients with late-stage knee osteoarthritis (KOA). **Methods**: A total of 17 (6M, 11F) patients (aged 69 ± 10 years) were assessed for peripheral (pressure pain thresholds (PPT)) and central (temporal summation (TS) and conditioned pain modulation (CPM)) sensitization the day before total knee arthroplasty. Synovial fluid was collected during surgery and assayed for IL-6, IL-8, IL-10, TNF-α, CXCL-10, BDNF, NGF, CCL2, CCL5, VEGF, IL-1RI, MMP-1, MMP-7, IL-1β, and CXCL-9. Associations of biomarkers and their combinations reflecting chronic (CXCL-9) and acute ((CCL2×CXCL-10)/IL-10)) inflammation, cartilage degeneration (MMP-1×MMP-7), and neurotrophy (NGF×BDNF) with PPT, TS, and CPM were analyzed by bivariate correlations and by multiple linear regression analyses corrected for BMI, sex, and age. **Results**: The medial joint line and the superior medial joint region showed the lowest PPT. Higher acute inflammation related significantly to worse pressure tenderness at the superior medial joint region (R^2^ = 0.642; *p* = 0.010). Cartilage degeneration and chronic inflammation were associated with both absolute (R^2^ = 0.827; *p* = 0.001) and relative CPM (R^2^ = 0.882; *p* < 0.001). Acute inflammation and neurotrophy were related to relative TS at the m. tibialis anterior (R^2^ = 0.728; *p* = 0.02). **Conclusions**: This study demonstrates that increased levels of intra-articular biomarkers of acute inflammation are related to peripheral sensitization and that biomarkers of cartilage degeneration and chronic inflammation are associated with central sensitization. These results may be a stepping-stone toward a better understanding of the working mechanism of peripheral and central sensitization in KOA pain and the development of more targeted therapeutic interventions.

## 1. Introduction

Knee pain is one of the most common symptoms in patients with knee osteoarthritis (KOA), leading to discomfort, disability, and decreased quality of life. Previous joint damage and inflammation (i.e., synovitis) are recognized as contributing factors in this pathogenesis [1]. Historically, osteoarthritis (OA)-related pain was assumed to be caused by structural damage in the knee joint and categorized as nociceptive pain [2]. However, severe radiographic knee damage does not necessarily result in important knee pain [3]. Late-stage KOA patients suffering from high knee pain levels and having a poor func-tionality frequently opt for a total knee arthroplasty (TKA) surgery to improve quality of life [4]. However, a minority of these patients have persistent pain after TKA. Recent research [5] shows that high preoperative pain levels and low levels of radiographic knee damage are associated with higher knee pain after TKA surgery. This indicates that knee tissue damage is not always a nociceptive source in KOA patients. Adaptations in peripheral and central neurophysiological mechanisms contribute to OA related pain [3]. Therefore, OA-related pain potentially adheres to the definition and clinical criteria of nociplastic pain defined by the International Association for the Study of Pain as “pain that arises from altered nociception with sensitization as the major underlying mechanism” [6]. Joint nociceptors can become sensitized because of, e.g., inflammation, leading to peripheral sensitization. Peripheral sensitization occurs when the excitation threshold of the nociceptors is reduced, and noxious and non-noxious input result in hyperalgesia and allodynia, respectively [3]. Sustained or chronic nociceptive signaling (e.g., inflammation) from the affected knee can induce central sensitization. In this case, spinal and supraspinal nociceptive mechanisms become sensitized, causing nociceptive facilitation. Additionally, nociceptive facilitation can result from an impairment in the descending nociceptive inhibitory pathway at the level of the dorsal horn. Furthermore, widespread allodynia and hyperalgesia are typical aspects in central-sensitized patients [3,7]. Features of human-assumed central sensitization are commonly observed in patients with KOA, fueling the idea of a nociplastic pain subgroup within the KOA population [8]. A series of quantitative sensory testing (QST) measures (e.g., pain thresholds, temporal summation (TS), and conditioned pain modulation (CPM)) allow investigation of the effectiveness of the pain modulation system in humans. One characteristic of peripheral sensitization is a decreased pain threshold, specifically at the level of the affected knee. On the other hand, in patients with human-assumed central sensitization, a decreased pain threshold is more generalized and also acts on regions outside the affected area. Furthermore, enhanced TS (a measure for pain facilitation) and/or an impaired CPM (a measure for endogenous analgesia) are two additional characteristics of human-assumed central sensitization [3,6,9].

A recent review of the literature emphasized that markers of inflammation, cartilage degeneration, and neurotrophic factors are potential mediators in the initiation of sensitization and, consequently, in OA-related pain [3]. Neurotrophic factors like brain-derived neurotrophic factor (BDNF) and nerve growth factor (NGF) can contribute to peripheral and central sensitization [3,10]. These factors are primarily responsible for the development and survival of neurons in the human nervous system. In addition to its principal functions within the nervous system, NGF plays a role in the immune response [11]. Specifically, mast cells could release pro-nociceptive mediators that contribute to the activation and/or sensitization of peripheral nociceptors. NGF is a mediator that activates TrkA expressed on nociceptors, leading to peripheral sensitization [11]. Given that NGF levels are high in the synovium of patients with KOA, pharmaceuticals are under development to either neutralize free NGF or inhibit the binding of NGF on its receptors to reduce OA-related pain [10,12]. Another biomarker group of interest that plays a role in sensitization in patients with OA is acute phase inflammatory mediators [3]. Synovitis, inflammation of the joint synovium, and systemic low-grade inflammation are key for KOA. Following joint injury or overload, an innate immune response is induced, and immune cells (e.g., macrophages) release inflammatory mediators into the joint cavity [3,13]. Crucial pro-inflammatory cytokines are interleukin (IL)-1β and tumor necrosis factor (TNF)-α, which further activate chondrocytes and fibroblasts to produce other markers like IL-6 and IL-8 and initiate matrix metalloproteinase (MMP) activity [12,14]. Some of these cytokines can directly activate nociceptors (as their receptors are expressed) and sensitize them. Others work indirectly by further sustaining the inflammatory response [3]. Additionally, chemokines attract immune cells to the inflamed tissue and promote inflammation. However, evidence from rodent studies showed that C-C motif ligand (CCL)2 is also involved in chronic pain as it can directly activate sensory neurons via its receptor CCR2 [3]. Future research should focus on the role of CCL2, but also of CCL5 and C-X-C motif ligand (CXCL)-10 in OA-related pain [14]. Toll-like receptors (TLRs) expressed on sensory neurons can be activated by damage-associated molecular pattern proteins (DAMPs), such as fragments of cartilage breakdown [3]. Levels of MMP-1 and MMP-7 are elevated in OA, with MMP-1 being associated with symptomatic OA [3,14]. Furthermore, it appears that MMP-13 (having a vital role in the cleavage of type II collagen) is a central player in the degradation of joint cartilage, along with MMP-7 (having collagen type I and IV as substrate) [15]. However, research on the function of MMP-7 in KOA pain is challenging [14], and literature data are scarce. Additionally, iAge, described as “a metric for age-related inflammation” [16], can also be considered in KOA because aging is one of the main risk factors for OA [1], and chronic inflammation is a hallmark of KOA [13]. Chemokine CXCL-9 has a significant contribution to iAge and could be a good biomarker for chronic inflammation in the context of aging [16].

The associations between biomarkers in the synovial fluid and parameters of sensitization have received limited attention in previous human KOA research despite their potential relevance. Most evidence on this topic originates from rodent studies [17]. Previous studies in OA patients have predominantly focused on systemic biomarkers or estimated joint inflammation using imaging methods [14,18,19,20,21,22]. However, recent recommendations [18,23] advocate for measuring intra-articular levels of biomarkers that reflect the state of the affected joint instead of blood-based circulating levels [18]. Therefore, we investigated the association of four groups of biomarkers (i.e., neurotrophic, joint inflammation, cartilage degeneration, and age-related inflammation) in synovial fluid of OA knees, with parameters of sensitization in this study.

## 2. Materials and Methods

### 2.1. Study Design

Baseline data of a subgroup of participants in a randomized controlled trial (RCT) [24] was used for this observational, exploratory study. The study was approved by the ethical committee of UZ Brussel (B.U.N. 14320108336) and was conducted according to the Declaration of Helsinki.

### 2.2. Setting and Participants

**Figure 1:**
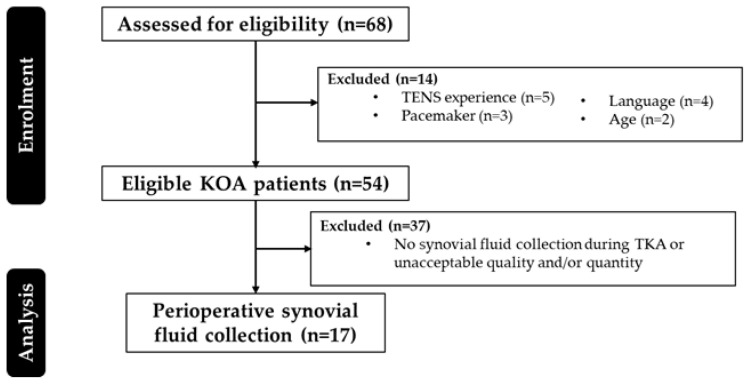
Participant flowchart. TENS: transcutaneous electrical neurostimulation; KOA: knee osteoarthritis; TKA: total knee arthroplasty.

In summary, and as reported previously [24], subjects were recruited between March 2013 and January 2015. Seventeen patients (≥50 years) diagnosed with KOA, according to the American College of Rheumatology criteria [25], were eligible (Figure 1). These patients had invalidating symptoms of KOA and failed to respond to conservative treatment for at least three months. They were scheduled for a TKA by two orthopedic surgeons after informed consent. Patients with prior use of transcutaneous electrical neurostimulation (TENS), contraindications for TENS, or proficiency with a TENS device were excluded, along with those who did not have proficiency in Dutch or French. Prior to enrollment, all participants provided informed consent.

### 2.3. Assessments

All baseline measurements were performed one day before TKA surgery, except the collection of synovial fluid, which was obtained during the surgery.

#### 2.3.1. Patient Characteristics

Personal and disease-related information (e.g., age, BMI, previous surgeries) was collected via questionnaires or extracted from the patients’ medical records.

#### 2.3.2. Knee Pain

To assess knee pain, an 11-point numeric rating scale (NRS) (0 = no pain; 10 = maximum pain) and the pain subscale of the knee injury and osteoarthritis outcome score (KOOS) questionnaire were used. An NRS score was obtained for the following seven scenarios: (1) pain at this moment, (2) maximum pain in the last 24 h, (3) minimum pain in the last 24 h, (4) maximum pain in the last 7 days, (5) minimum pain in the last 7 days, (6) maximum pain in the last 30 days, and (7) minimum pain in the last 30 days. The KOOS evaluates OA-related symptoms and limitations. It contains five domains: pain (9 items), symptoms (7 items), functioning in daily life (17 items), functioning in leisure and sports (5 items), and knee-related quality of life (4 items) [26]. If 1 to 3 items were not scored in the first four domains, the overall score per domain was rescaled. If more than 3 items were missing in these domains, the data were considered missing. For the ‘knee-related quality of life’ domain, data were considered missing if <50% of the questions were unanswered.

#### 2.3.3. Characteristics of Peripheral and Central Sensitization

**Figure 2:**
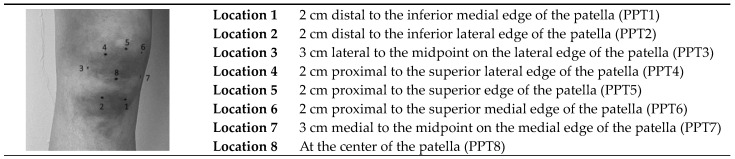
Eight PPT measurement locations around the knee. PPT: pressure pain threshold.

Pressure pain thresholds (PPTs) were determined using a handheld algometer (Somedic AB, Farsta, Stockholm, Sweden) according to the protocol of Arendt–Nielsen et al. [9]. There were eight test sites around the knee (Figure 2), and two control sites: one at the m. tibialis anterior (mta) and one at the m. extensor carpi radialis longus (crl). The pressure was increased at +/− 1 kg/s until the participant experienced the pressure as painful. The PPT assessment was carried out twice per test and control site.

Two minutes later, the TS assessment was performed at the test site with the lowest PPT and at the mta to obtain information about the pain facilitation system. Ten pressure pulses were applied via the handheld algometer at mean PPT intensity. The rate of each pressure pulse was increased by approximately 2 kg/s and maintained for 1 s. Pain levels were scored at the first, fifth, and tenth pulse. The absolute TS effect was calculated by [NRS10th pulse − NRS1rst pulse], and the relative TS effect by: TSrelative = [(NRS10th pulse − NRS1rst pulse)/NRS1rst pulse] × 100 [27].

The pain inhibition system was evaluated through the CPM paradigm, where ischemic compression served as the conditioning stimulus and pressure as the test stimulus. A tourniquet cuff was applied and inflated on the left arm until painful. Participants were then instructed to make a fist at least ten times until a pain score of 4 was reached. Subsequently, the left arm was rested, and the TS protocol was repeated. The absolute and relative CPM effects were calculated: CPMrelative = [(NRS10th pulse, tourniquet − NRS10th pulse, without tourniquet)/NRS10th pulse, without tourniquet] × 100 and CPMabsolute = [NRS10th pulse, tourniquet − NRS10th pulse, without tourniquet] [28]. A negative CPM score reflects pain inhibition, while a positive CPM score indicates pain facilitation [27].

#### 2.3.4. Synovial Fluid Collection and Biomarker Assays

Synovial fluid was collected via aspiration of the affected knee during TKA surgery and stored at −80 °C. All biomarkers were determined by a custom-made human multiplex assay (GeniePlex by AssayGenie, Dublin, Ireland). An 11-plex kit was used to analyze IL-6, IL-8, IL-10, TNF-α, CXCL-10, BDNF, NGF, CCL2, CCL5, VEGF, and IL-1RI, a 3-plex kit for MMP-1, MMP-7, and IL-1β, and a single-plex assay for CXCL-9. The sensitivity, lower limit of quantification, and upper limit of quantification of all analytes are reported in Supplementary Table S1.

### 2.4. Statistical Analysis

Statistical analyses were performed using Statistical Package of the Social Sciences (SPSS) version 29 (IBM, Amonk, New York, NY, USA). Descriptive statistics were applied for all outcomes, and means and standard deviations (SD) for normally distributed variables. For biomarkers not normally distributed, a log10 transformation was performed. An independent *t*-test was used to assess sex differences. Pearson correlations were calculated to assess the relation between the biomarker and sensitization levels. Univariate or multiple linear regressions, including stepwise regression with backward elimination, were used to assess the association between sensitization parameters (PPT, TS, CPM) and intra-articular biomarker levels. For the regression model, independent outcomes were: log(CXCL-9) reflecting chronic inflammation, and three composite factors reflecting acute inflammation (log(CXCL-10×CCL2/IL-10)), cartilage degeneration (log(MMP-1×MMP-7)), and neurotrophy (log(BDNF×NGF)). The biomarkers included in the composite outcomes were selected based on their bivariate association with the sensitization outcomes. Composite outcomes were used because a single biomarker might be insufficiently informative. All regression models were adjusted for BMI, sex, and age.

## 3. Results

### 3.1. Participants’ Characteristics

Seventeen participants (11 women and 6 men) were included. All participants had persistent knee pain (reflected by NRS scores > 3) and also experienced pain-related disability (reflected by KOOS scores > 55) (Table 1). Women showed a significantly higher BMI (*p* = 0.032), lower PPT at mta. (*p* = 0.046), higher intra-articular VEGF-A (*p* = 0.040), higher maximal pain levels in the last 24 h (*p* = 0.046), and worse functioning in recreation and sport (KOOS; *p* = 0.046) (Table 1). No other significant sex differences were observed. 

### 3.2. Bivariate Associations between Intra-Articular Biomarker Levels and Pain Sensitization Parameters

**Figure 3:**
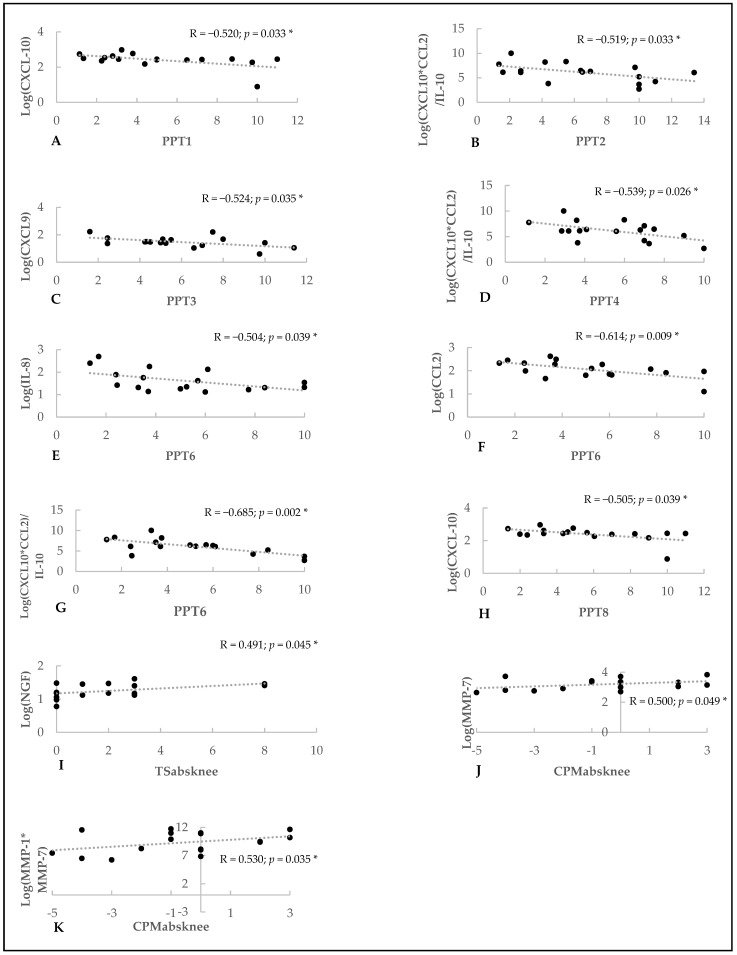
Bivariate associations between intra-articular biomarker levels and pain sensitization parameters. (**A**) Bivariate association between PPT1 and Log(CXCL-10). (**B**) Bivariate association between PPT2 and the predictor for acute inflammation. (**C**) Bivariate association between PPT3 and the predictor for chronic inflammation. (**D**) Bivariate association between PPT4 and the predictor for acute inflammation. (**E**) Bivariate association between PPT6 and Log(IL-8). (**F**) Bivariate association between PPT6 and Log(CCL2). (**G**) Bivariate association between PPT6 and acute inflammation. (**H**) Bivariate association between PPT8 and Log(CXCL-10). (**I**) Bivariate association between TSabsknee and Log(NGF). (**J**) Bivariate association between CPMabsknee and Log(MMP-7). (**K**) Bivariate association between CPMabsknee and the predictor for cartilage degeneration. * *p* < 0.05; R = Pearson correlation coefficient; PPT: pressure pain threshold; TS: temporal summation; abs: absolute; CPM: conditioned pain modulation; IL: interleukin; NGF: nerve growth factor; CCL: C-C motif ligand; CXCL: C-X-C motif ligand; MMP: matrix metalloproteinase.

As shown in Figure 3A–K, higher levels of the composite parameter reflecting acute inflammation (CXCL-10×CCL-2/IL-10) were significantly associated with lower PPTs at three sites around the knee, PPT2 (Pearson’s r: −0.519; *p* = 0.033), PPT4 (Pearson’s r: −0.539; *p* = 0.026), and PPT6 (Pearson’s r: −0.685; *p* = 0.002). Lower PPT at the superior medial joint region (PPT6) was significantly related to higher levels of IL-8 (Pearson’s r: −0.504; *p* = 0.039) and CCL-2 (Pearson’s r: −0.614; *p* = 0.009). Higher CXCL-10 levels were significantly associated with lower PPT1 (Pearson’s r: −0.520; *p* = 0.033) and PPT8 (Pearson’s r: −0.505; *p* = 0.039). Higher levels of chronic inflammation, reflected by CXCL-9, were significantly associated with lower PPT at location 3 (Pearson’s r: −0.514; *p* = 0.035). Increased pain facilitation at the affected knee joint (reflected by CPMabs) was significantly related to higher levels of cartilage degeneration biomarkers (MMP7 (Pearson’s r: 0.500; *p* = 0.049) and MMP1×MMP7 (Pearson’s r: 0.530; *p* = 0.035)). Finally, higher intra-articular NGF levels related significantly to increased TS at the affected knee (TSabs) (Pearson’s r: 0.491; *p* = 0.045). No significant associations were found between intra-articular levels of neurotrophy biomarkers (BDNF, NGF, BDNF×NGF) and any of the sensitization outcomes.

### 3.3. Peripheral and Central Sensitization Prediction by Intra-Articular Biomarker Levels

Multiple linear regression analyses were performed for each of the peripheral and central sensitization parameters (as dependent variables) and the four (composite) biomarker predictors (as independent variables). Several significant regression models were found (Table 2, Table 3, Table 4 and Table 5). The composite parameter reflecting acute inflammation (log(CXCL-10×CCL2/IL-10) was associated with lower PPT at the superior medial joint region of the affected knee (PPT6) (R^2^ = 0.642; *p* = 0.010). Both CPMrel, knee and CPMabs, knee were associated with the composite parameter reflecting cartilage degeneration (Log(MMP-1×MMP-7)) and chronic inflammation (Log(CXCL-9)), (R^2^ = 0.827; *p* = 0.001 and R^2^ = 0.882; *p* < 0.001, respectively). The composite parameters indicating acute inflammation, log(CXCL-10×CCL2/IL-10) and neurotrophy, log(BDNF×NGF), explained 72.8% of the TSrel, mta variance (R^2^ = 0.728; *p* = 0.020).

## 4. Discussion

This exploratory pilot study examined the relationship of intra-articular biomarkers of inflammation, neurotrophy, and cartilage degeneration with characteristics of human-assumed peripheral and central sensitization in seventeen patients with late-stage KOA. We found that increased levels of acute inflammatory biomarkers ((CXCL-10×CCL-2)/IL-10) in the synovial fluid are associated with lower PPT at the superior medial joint region of the affected knee (a feature of peripheral sensitization). Moreover, CPM (a feature of human-assumed central sensitization) in patients with late-stage KOA was related to higher intra-articular levels of cartilage degeneration (MMP-1×MMP-7) and chronic inflammation (CXCL-9).

The relationship between intra-articular cytokines and sensitization is previously investigated in rodent studies [17]. However, in those studies, the main focus was placed on four pro-inflammatory cytokines (TNF-α, IL-6, IL-1β and IL-17) [17]. Biomarkers reflecting cartilage degeneration and neurotrophy received limited attention [17,29]. The role of these biomarker groups should not be underestimated as we found that biomarkers of cartilage degeneration (MMP-1×MMP-7) and chronic inflammation (CXCL-9) are associated with central sensitization in humans with KOA. Schaible et al. even suggested to investigate the association of pain with cytokines in the brain. We found a positive correlation between intra-articular NGF levels and TS at the affected knee. The main conclusion of the review of Schaible et al., was that intra-articular biomarkers work directly on nociceptors and cause sensitization. This conclusion can be confirmed by our results in human KOA patients. To our knowledge, our study is the first that investigated the relationship between features of peripheral and central sensitization and intra-articular markers of inflammation in human patients with late-stage KOA.

The two most painful spots on the affected knee were located on the medial joint line (PPT7) and at the superior medial region (PPT6). Both are located on the medial site of the knee, where patients with KOA usually experience the most pain [30,31]. However, literature data about pain in the superior medial area of the knee are more heterogeneous [30,31]. The arthroscopic study by Ayral et al. concluded that inflammation of the medial perimeniscal synovium was related to higher pain scores and more serious chondral lesions [32]. As such, it can be hypothesized that increased levels of inflammation in the synovial fluid (i.e., presence of synovitis) contribute to the development of a higher sensitivity for pain on the medial site of the knee. Additionally, Neogi et al. demonstrated an inverse relationship between PPT and synovitis. However, in that study, synovitis was estimated based on the Whole-Organ Magnetic Resonance Imaging Score [20].

Our results showed that a lower PPT at the superior medial region of the knee was significantly correlated with higher levels of IL-8 and CCL-2, and the composite parameter reflected acute inflammation. This aligns with a previous study that showed that synovial CCL2 is associated with higher self-reported pain and physical disability in KOA patients [33]. Recently, an experimental KOA mice model showed that intra-articular CCL2-CCR2 signaling is responsible for knee hyperalgesia assessed by the application of pressure [34]. This is largely accomplished by binding directly the intra-articular CCR2 that is expressed on sensory afferents located in the synovial membrane. Blocking the CCL2-CCR2 signaling was recognized as a therapeutic target to reduce knee hyperalgesia [32]. Also, the anti-inflammatory IL-10 receives attention as a potential therapeutic target to reduce inflammation and OA pain [35]. Synovial IL-10 can counteract the action of pro-inflammatory cytokines, and can provoke chondroprotective effects by blocking MMP activity [35,36]. Nees et al. [36] did observe a correlation between synovial IL-10 and subjective knee pain. Surprisingly, in the study by Imamura et al. [37], no association was found between serum IL-10 and PPT measures in KOA patients [37]. Although not completely the same KOA patients were included as in the paper of Imamura et al. [37], our study population showed several similarities including age ≥ 60 years, KOA diagnosis based on ACR criteria and knee pain. However, our study population showed end-stage KOA (versus K&L grade 2 or higher), which may explain partly this discrepancy. 

The influence of CXCR3, and its ligand CXCL-10, should not be underestimated in the modulation of pain [38]. CXCR3-CXCL-10 signaling is responsible for the recruitment of immune cells to the site of inflammation (i.e. synovium), and inflammatory mediators can stimulate primary sensory afferents in the synovial membrane [38]. CXCL-10 produced by neutrophils recruits NK cells and macrophages in the synovial fluid. Both cells can stimulate OA progression. A pre-clinical study [39] reported that CXCR3 – CXCL-10 signaling is crucial in the pathogenesis of OA because CXCR3 knock-out mice did not develop OA and had no elevated synovial CXCL-10. Furthermore, CXCR3 – CXCL-10 signaling contributes to descending nociceptive pain facilitation [38]. Descending pain modulation in the dorsal horn was investigated in a mouse model with joint pain [40]. A reduction in µ-opioid receptors (MOR) expressed on ON-cells in the rostroventromedial medulla induced a downregulation of CXCR3, CXCL-9 and CXCL-10 genes in the dorsal horn. It was observed that a decline in these MOR expressing cells also weakened the pain hypersensitivity [40]. As such, CXCR3 can also be a potential target to reduce peripheral and central sensitization [38,40].

We found that dysfunctional endogenous nociceptive modulation measured using the CPM paradigm, a feature of central sensitization, was associated with intra-articular concentrations of MMP-1, MMP-7, and CXCL-9. This means that when the levels of these biomarkers are higher, knee pain is facilitated instead of inhibited. Unexpectedly, in the multiple regression model, CXCL-9, a marker of chronic inflammation, showed a negative β coefficient. In fact, circulating CXCL-9 in blood serum is an excellent biomarker for capturing aging-related chronic inflammation [16]. In the same regression model, MMP-1 and MMP-7 related positively to CPM. The expression of MMP-1 is increased in the synovium in the case of OA, and MMP-1 levels in the synovial fluid are significantly higher in advanced KOA [41]. MMP-7 is scarcely investigated in OA. Nevertheless, it has a meaningful contribution to the pathogenesis of OA and is overexpressed in OA chondrocytes [15,42]. When there is an excess of catabolism and a deficiency of anabolism of the cartilage extracellular matrix, OA can be promoted [43]. Notwithstanding, higher levels of cartilage degradation are not always associated with higher pain levels in OA patients [44]. According to Anitua et al. [45], there is no relationship between synovial MMP concentrations and radiographic severity in OA patients [45]. In a subpopulation of patients with KOA, human-assumed central sensitization is present [46]. The majority of OA patients with human-assumed central sensitization has no severe tissue damage on radiographs but has high pain intensity scores, because the endogenous analgesic pain modulatory system is not functioning well [3]. When looking into the CPM levels of our participants, a high standard deviation was observed pointing to a high variability. Hence, it may indicate that the descending nociceptive inhibitory pathway is malfunctioning in some of the KOA participants, causing pain facilitation. Our model demonstrated that 82.7% of the variance in CPM of the affected knee could be explained by MMP-1×MMP-7 and CXCL-9. However, there is still some variance that is not declared meaning that we missed some other interested biomarkers, for instance tissue inhibitors of metalloproteinases (TIMPs), that can be investigated in future research.

Remarkably, 73% of the variability in TS at the mta could be explained by the levels of acute inflammation and neurotrophy. However, we were unable to find a similar association at the level of the affected knee. The test location on the mta is a region located outside the affected knee area and can be considered as non-injured, and indicative for human-assumed central sensitization [9]. Nociceptive stimulation of a muscle, may lead to the formation of new receptive areas of the neuron [47]. It is suggested that neurons of contiguous spinal segments also become sensitized because of sensitization spreading. As such, it can be assumed that intra-articular biomarkers of inflammation and neurotrophic factors contribute to spreading (central) sensitization to the mta.

QST has been used for more than a decade in OA pain research [48,49], however, its application in exploring the role of intra-articular biomarkers in human-assumed central and peripheral sensitization is innovative. The merits of QST are mainly that it provides valuable information on the working of the somatosensory and pain modulation system [50,51]. However, this method has also a few shortcomings, as it is a time-consuming procedure and the equipment is expensive. Future research needs to focus on the development of guidelines or approaches to classify patients with pain into different subgroups based on their pain typology which can be determined by QST. As such, targeted therapies can be developed for each pain subgroup [50].

Sex differences in this KOA pain population were observed, which is not uncommon according to literature [52]. Previous studies indicated that women experience more pain than men, and suffer more from functional impairments [52]. We found a significant difference in the maximum pain experienced in the last 24 hours, with women reporting significantly more pain than men. Additionally, women scored significantly higher in KOOS functioning in recreation and sports. Sex hormones (i.e., estrogens and testosterone) may explain this observation [52]. An additional explanation could be the difference in BMI between women and men in our study. Specifically, five men were categorized as overweight (BMI 25–30 kg/m^2^) and one as obese (BMI ≥ 30kg/m^2^), while five women were overweight and six were obese. In a study by Raud et al. [53], obese KOA patients, experienced more often pain in the last 24 hours than KOA patients with overweight [53], and BMI was associated with the severity of KOA related pain [53]. Overweight or obesity are also key risk factors for in the development of OA because of local (i.e. increased joint loading) and systemic effects (i.e., metabolic diseases). An increase of 5-units in BMI results in an 35% increase in the risk to develop OA [53]. Furthermore, overweight and obesity are associated with disability, which can also explain the difference in the KOOS recreation and sports functioning score [53]. Another remarkable finding was a significant difference in synovial VEGF-A, with higher levels in women than in men. This finding aligns with the results of Kisand et al. [54]. They reported a positive correlation between OA and cytokines involved in angiogenesis in women with an early stage of KOA [54]. 

This study has some limitations. The sample size was small as it was an exploratory add-on study of a RCT. In the future, a larger cross-sectional study is necessary before making firm conclusions. Unfortunately, we have not diagnosed the presence of knee synovitis by histology, MRI or U-sound. Furthermore, we suggest to examine also other interesting biomarkers in the synovial fluid (e.g., TIMPs). This study has some strengths. Biomarker analyses were performed with an advanced technique, allowing multiple intra-articular biomarker dosage with a small amount of fluid (i.e. 15 µL). Collection of synovial fluid is challenging because blood contamination will lead to hemolysis which will compromise the sample, and maximum 3.5 mL of synovial fluid can be obtained [55]. We were able to collect synovial fluid of 17 participants. It cannot be excluded that those subjects with less pronounced synovitis might have shown insufficient synovial fluid to collect in this study, which might have introduced some selection bias. It is one of the first studies that reports on the relationship between intra-articular biomarkers and characteristics of human-assumed peripheral and central sensitization in KOA patients. Our results suggest that joint inflammation and human-assumed peripheral and central sensitization are closely related. This is a stepping-stone towards the unraveling of the working mechanisms of peripheral and central sensitization in KOA pain patients. It is important to investigate that relationship and unravel the working mechanism of knee pain in human OA. On the other hand, it could allow us to identify intra-articular biomarkers that may predict and/or influence sensitization in KOA patients. This could open doors towards new therapeutic approaches for (non-)sensitized patients with KOA (e.g., targeting specific biomarkers) [56].

## Figures and Tables

**Table 1:** Patients’ characteristics.

	*n*	Global (Mean ± SD)	*n*	Women (Mean ± SD)	*n*	Men (Mean ± SD)	*p*-Value
Demographics:							
Age (years)	17	68.59 ± 10.04	11	70.45 ± 10.05	6	65.17 ± 10.89	0.541
BMI (kg/m^2^)	17	31.40 ± 5.08	11	32.93 ± 5.94	6	28.61 ± 1.67	0.032 *
Parameters of pain sensitization:							
PPT1 (kg/s)	17	5.16 ± 3.16	11	4.20 ± 2.67	6	6.93 ± 3.46	0.231
PPT2 (kg/s)	17	6.39 ± 3.75	11	5.89 ± 3.89	6	7.30 ± 3.62	0.940
PPT3 (kg/s)	17	5.92 ± 2.76	11	5.82 ± 3.17	6	6.10 ± 2.06	0.134
PPT4 (kg/s)	17	5.38 ± 2.44	11	5.01 ± 2.12	6	6.05 ± 3.04	0.117
PPT5 (kg/s)	17	6.22 ± 3.13	11	6.05 ± 3.03	6	6.53 ± 3.58	0.399
PPT6 (kg/s)	17	5.08 ± 2.71	11	4.35 ± 2.54	6	6.41 ± 2.71	0.651
PPT7 (kg/s)	17	4.22 ± 2.15	11	3.40 ± 1.77	6	5.72 ± 2.08	0.926
PPT8 (kg/s)	17	5.66 ± 3.06	11	4.51 ± 2.08	6	7.77 ± 3.61	0.063
PPT mta (kg/s)	17	6.66 ± 2.98	11	6.14 ± 2.99	6	7.62 ± 2.96	0.851
PPT crl (kg/s)	17	5.30 ± 2.96	11	4.35 ± 1.99	6	7.05 ± 3.80	0.046 *
TS _abs knee_ (kg/s)	17	2.47 ± 2.90	11	2.45 ± 2.98	6	2.50 ± 3.02	0.960
TS _abs mta_ (kg/s)	17	3.24 ± 3.35	11	3.09 ± 3.51	6	3.50 ± 3.33	0.862
CPM_abs knee_ (kg/s)	16	−0.69 ± 2.47	10	−0.40 ± 2.91	6	−1.17 ± 1.60	0.183
CPM_abs mta_ (kg/s)	17	0.47 ± 2.60	11	0.91 ± 3.08	6	−0.33 ± 1.21	0.253
CPM_rel knee_ (kg/s)	16	−3.90 ± 59.86	10	10.43 ± 67.23	6	−27.78 ± 38.97	0.095
CPM_rel mta_ (kg/s)	17	42.65 ± 155.91	11	68.85 ± 186.11	6	−5.37 ± 65.06	0.147
Biomarkers (pg/mL):							
Log IL-6	17	2.04 ± 0.67	11	2.29 ± 0.52	6	1.58 ± 0.72	0.699
Log IL-8	17	1.62 ± 0.48	11	1.70 ± 0.53	6	1.49 ± 0.38	0.253
Log TNF-α	17	0.43 ± 0.55	11	0.52 ± 0.59	6	0.25 ± 0.46	0.437
Log NGF	17	1.26 ± 0.22	11	1.30 ± 0.18	6	1.20 ± 0.28	0.483
Log BDNF	17	1.38 ± 0.32	11	1.46 ± 0.25	6	1.24 ± 0.41	0.126
Log CCL-5	17	0.69 ± 0.89	11	0.76 ± 0.86	6	0.57 ± 1.00	0.820
Log IL1-R1	17	1.71 ± 0.29	11	1.79 ± 0.27	6	1.58 ± 0.31	0.942
Log VEGF-A	17	1.73 ± 0.35	11	1.83 ± 0.19	6	1.54 ± 0.51	0.040 *
Log CCL-2	17	2.06 ± 0.37	11	2.16 ± 0.28	6	1.89 ± 0.47	0.349
Log CXCL-10	17	2.40 ± 0.44	11	2.53 ± 0.21	6	2.15 ± 0.63	0.083
Log IL-10	17	0.85 ± 0.26	11	0.92 ± 0.21	6	0.74 ± 0.33	0.276
Log IL-1β	17	0.55 ± 0.51	11	0.49 ± 0.59	6	0.65 ± 0.34	0.174
Log MMP-1	17	2.90 ± 0.39	11	2.93 ± 0.43	6	2.84 ± 0.31	0.513
Log MMP-7	17	3.19 ± 0.36	11	3.25 ± 0.35	6	3.08 ± 0.39	0.597
Log CXCL-9	17	1.47 ± 0.40	11	1.51 ± 0.47	6	1.39 ± 0.20	0.103
Predictors:							
Acute inflammation: Log(CXCL10×CCL2)/IL-10	17	6.15 ± 1.87	11	6.19 ± 1.42	6	6.07 ± 2.67	0.088
Cartilage degeneration: Log(MMP1×MMP7)	17	9.28 ± 1.89	11	9.55 ± 1.95	6	8.79 ± 1.82	0.888
Neurotrophy: Log(BDNF×NGF)	17	1.81 ± 0.65	11	1.93 ± 0.55	6	1.58 ± 0.80	0.420
Chronic inflammation: Log(CXCL9)	17	1.47 ± 0.40	11	1.51 ± 0.47	6	1.39 ± 0.20	0.103
Knee pain scores (questionnaires):							
NRS 1 _at this moment_	15	4.80 ± 2.57	9	4.56 ± 2.92	6	5.17 ± 2.14	0.263
NRS 2 _max. pain ≤ 24 h_	15	6.67 ± 2.16	9	7.56 ± 1.33	6	5.33 ± 2.58	0.046 *
NRS 3 _min. pain ≤ 24 h_	15	3.93 ± 2.40	9	4.33 ± 2.83	6	3.33 ± 1.63	0.100
NRS 4 _max. pain ≤ 7 d_	15	6.60 ± 2.13	9	7.22 ± 1.79	6	5.67 ± 2.42	0.201
NRS 5 _min. pain ≤ 7 d_	15	3.80 ± 2.46	9	4.00 ± 2.96	6	3.50 ± 1.64	0.274
NRS 6 _max. pain ≤ 30 d_	15	7.07 ± 2.43	9	7.78 ± 2.33	6	6.00 ± 2.37	0.498
NRS 7 _min. pain ≤ 30 d_	15	3.40 ± 2.13	9	3.33 ± 2.50	6	3.50 ± 1.64	0.286
KOOS _pain_	14	62.40 ± 22.48	9	71.76 ± 13.18	5	45.56 ± 27.26	0.087
KOOS _symptoms_	15	55.24 ± 14.59	9	57.94 ± 15.75	6	51.19 ± 12.91	0.706
KOOS _functioning in daily life_	13	56.93 ± 23.09	8	69.32 ± 13.38	5	48.49 ± 30.87	0.320
KOOS _functioning in recreation & sport_	8	66.25 ± 43.16	4	93.75 ± 12.5	4	38.75 ± 46.61	0.046 *
KOOS _Qol_	15	82.59 ± 10.74	9	82.64 ± 12.01	6	68.75 ± 34.69	0.135

* *p* < 0.05. SD: standard deviation; BMI: body mass index; PPT: pressure pain threshold; TS: temporal summation; abs: absolute; rel: relative; CPM: conditioned pain modulation; mta: musculus tibialis anterior; crl: m. ext. carpi radialis longus; IL: interleukin; TNF: tumor necrosis factor; NGF: nerve growth factor; BDNF: brain-derived neurotrophic factor; CCL: C-C motif ligand; CXCL: C-X-C motif ligand; VEGF: vascular endothelial growth factor; MMP: matrix metalloproteinase; NRS: numeric rating score; KOOS: knee injury and osteoarthritis outcome score; Qol: quality of life.

**Table 2:** Regression model for PPT at location 6 at the knee.

R^2^ = 0.642; *p* = 0.010 *	β Coefficients	*p* Value	95% Confidence Interval for β	
PPT6			Lower Bound	Upper Bound
(Constant)	13.844	0.066	−1.056	28.743
Acute inflammation: Log((CXCL10× CCL2)/IL-10)	−1.030	0.002	−1.603	−0.453
Age	−0.055	0.318	−0.170	0.060
Gender	1.744	0.156	−0.767	4.255
BMI	0.023	0.837	−0.214	0.260

* *p* < 0.05. BMI: body mass index; PPT: pressure pain threshold; IL: interleukin; CCL: C-C motif ligand; CXCL: C-X-C motif ligand; R^2^: coefficient of determination; β: regression coefficient.

**Table 3:** Regression model for CPM_relative_ measured at the knee.

R^2^ = 0.827; *p* = 0.001 *	β Coefficients	*p* Value	95% Confidence Interval for β	
CPM_rel, knee_			Lower Bound	Upper Bound
(Constant)	−125.911	0.300	−382.319	130.496
Cartilage degeneration: Log((MMP-1×MMP-7)	24.076	<0.001	12.604	35.548
Chronic inflammation: Log(CXCL-9)	−102.429	0.006	−168.020	−36.839
Age	−0.680	0.644	−3.860	2.501
Gender	−14.803	0.462	−57.951	28.345
BMI	3.071	0.178	−1.654	7.797

* *p* < 0.05. BMI: body mass index; rel: relative; CPM: conditioned pain modulation; CXCL: C-X-C motif ligand; MMP: matrix metalloproteinase; R^2^: coefficient of determination; β: regression coefficient.

**Table 4:** Regression model for CPM_absolute_ measured at the knee.

R^2^ = 0.882; *p* < 0.001 *	β Coefficients	*p* Value	95% Confidence Interval for β	
CPM_abs, knee_			Lower Bound	Upper Bound
(Constant)	−11.335	0.016	−20.065	−2.604
Cartilage degeneration: Log((MMP-1×MMP-7)	0.968	<0.001	0.577	1.359
Chronic inflammation: Log(CXCL-9)	−4.321	0.002	−6.554	−2.087
Age	−0.024	0.629	−0.133	0.084
Gender	0.939	0.185	−0.531	2.408
BMI	0.290	0.002	0.129	0.451

* *p* < 0.05. BMI: body mass index; abs: absolute; CPM: conditioned pain modulation; CXCL: C-X-C motif ligand; MMP: matrix metalloproteinase; R^2^: coefficient of determination; β: regression coefficient.

**Table 5:** Regression model for TS_relative_ measured at m. tibialis anterior.

R^2^ = 0.728; *p* = 0.020 *	β Coefficients	*p* Value	95% Confidence Interval for β	
TS_rel, mta_			Lower Bound	Upper Bound
(Constant)	−892.033	0.331	−2854.948	1070.882
Acute inflammation: Log((CXCL-10×CCL-2)/IL-10)	175.517	0.005	66.988	284.046
Neurotrophy: Log(BDNF×NGF)	651.170	0.002	314.676	987.665
Age	−11.238	0.132	−26.589	4.113
Gender	−128.661	0.430	−481.048	223.726
BMI	−13.538	0.316	−42.387	15.310

* *p* < 0.05. BMI: body mass index; TS: temporal summation; rel: relative; mta: musculus tibialis anterior; IL: interleukin; NGF: nerve growth factor; BDNF: brain-derived neurotrophic factor; CCL: C-C motif ligand; CXCL: C-X-C motif ligand; R^2^: coefficient of determination; β: regression coefficient.

## Data Availability

The data supporting the conclusions of this article will be made available by the authors upon request.

## Supplementary Materials

The following supporting information can be downloaded at https://www.mdpi.com/article/10.3390/jcm13175212/s1, Table S1: Overview of sensitivity, LLOQ, and ULOQ for each biomarker included in the GeniePlex.
