# Supplementary material for: Linking Intra-Articular Inflammatory Biomarkers with Peripheral and Central Sensitization in Late-Stage Knee Osteoarthritis Pain: A Pilot Study
Source: J Clin Med. 2024 Sep 2;13(17):5212. doi: 10.3390/jcm13175212 (PMC11395782; doi:10.3390/jcm13175212)
Supplement: Supplementary file 1 [file jcm-13-05212-s001.zip › jcm-3111438-supplementary.pdf]

Supplementary information

Supplementarty Tabel S1: Overview of sensitivity, LLOQ and ULOQ for each biomarker included into the GeniePlex.

Biomarker	Sensitivity (pg/mL)	LLOQ (pg/mL)	ULOQ (pg/mL)
IL-1β	<5	<10	<5 000
IL-6	<5	<10	<5 000
IL-8	<1	<1	<1 000
IL-10	<2	<5	<5 000
TNF-α	<1	<2	<1 000
CXCL10	<3	<10	<2 000
CCL2	<2	<5	<5 000
CCL5	<5	<10	<5 000
IL-1RI	<20	<50	<10 000
MMP-1	<100	<200	<20 000
MMP-7	<100	<200	<20 000
VEGF	<2	<5	<2 000
NGF	<5	<10	<5 000
BDNF	<5	<10	<5 000
CXCL9	<5	<10	<5 000

IL: interleukin; TNF: tumor necrosis factor; NGF: nerve growth factor; BDNF: brain-derived neurotrophic factor; CCL: C-C motif ligand; CXCL: C-X-C motif ligand; VEGF: vascular endothelial growth factor; MMP: matrix metalloproteinase; LLOQ: lower limit of quantification; ULOQ: upper limit of quantification
